# Renal scar formation and kidney function following antibiotic-treated murine pyelonephritis

**DOI:** 10.1242/dmm.030130

**Published:** 2017-11-01

**Authors:** Patrick D. Olson, Lisa K. McLellan, Alice Liu, Kelleigh L. Briden, Kristin M. Tiemann, Allyssa L. Daugherty, Keith A. Hruska, David A. Hunstad

**Affiliations:** 1 Medical Scientist Training Program, Washington University School of Medicine, St Louis, MO 63110, USA; 2 Department of Pediatrics, Washington University School of Medicine, St Louis, MO 63110, USA; 3 Department of Cell Biology and Physiology, Washington University School of Medicine, St Louis, MO 63110, USA; 4 Department of Molecular Microbiology, Washington University School of Medicine, St Louis, MO 63110, USA

**Keywords:** Pyelonephritis, Renal scarring, Fibrosis, Urinary tract infection, Chronic kidney disease, Hydronephrosis

## Abstract

We present a new preclinical model to study treatment, resolution and sequelae of severe ascending pyelonephritis. Urinary tract infection (UTI), primarily caused by uropathogenic *Escherichia coli* (UPEC), is a common disease in children. Severe pyelonephritis is the primary cause of acquired renal scarring in childhood, which may eventually lead to hypertension and chronic kidney disease in a small but important fraction of patients. Preclinical modeling of UTI utilizes almost exclusively females, which (in most mouse strains) exhibit inherent resistance to severe ascending kidney infection; consequently, no existing preclinical model has assessed the consequences of recovery from pyelonephritis following antibiotic treatment. We recently published a novel mini-surgical bladder inoculation technique, with which male C3H/HeN mice develop robust ascending pyelonephritis, highly prevalent renal abscesses and evidence of fibrosis. Here, we devised and optimized an antibiotic treatment strategy within this male model to more closely reflect the clinical course of pyelonephritis. A 5-day ceftriaxone regimen initiated at the onset of abscess development achieved resolution of bladder and kidney infection. A minority of treated mice displayed persistent histological abscess at the end of treatment, despite microbiological cure of pyelonephritis; a matching fraction of mice 1 month later exhibited renal scars featuring fibrosis and ongoing inflammatory infiltrates. Successful antibiotic treatment preserved renal function in almost all infected mice, as assessed by biochemical markers 1 and 5 months post-treatment; hydronephrosis was observed as a late effect of treated pyelonephritis. An occasional mouse developed chronic kidney disease, generally reflecting the incidence of this late sequela in humans. In total, this model offers a platform to study the molecular pathogenesis of pyelonephritis, response to antibiotic therapy and emergence of sequelae, including fibrosis and renal scarring. Future studies in this system may inform adjunctive therapies that may reduce the long-term complications of this very common bacterial infection.

## Abstract (precis)

**Summary:** A new model of antibiotic-treated severe pyelonephritis offers a novel platform to study the molecular pathogenesis of pyelonephritis, response to antibiotic therapy, and sequelae, including fibrosis and renal scarring.

## INTRODUCTION

Urinary tract infection (UTI) is a common affliction across the human lifespan, regularly affecting infants and young children in the first years of life (Foxman, 2003, 2010; Foxman and Brown, 2003; Arshad and Seed, 2015). Ascension of uropathogens to the kidneys can lead to pyelonephritis, which, even with successful antibiotic treatment, may carry long-term repercussions for the patient, including the development of renal scarring, hypertension and eventual progression to end-stage renal disease (Jacobson et al., 1989; Martinell et al., 1996; Wennerström et al., 2000; Levey and Coresh, 2012). Ascending bacterial infection of the renal parenchyma in humans elicits severe tubulointerstitial inflammation (Goluszko et al., 1997; Svensson et al., 2005, 2011; Mak and Kuo, 2006; Olson et al., 2016; Li et al., 2017). This innate inflammatory response, perhaps as much as bacterial processes *per se*, may largely underlie renal damage resulting from UTI, and is correlated with loss of functional renal tissue (scarring) and the development of fibrosis (Miller and Phillips, 1981; Bille and Glauser, 1982; Anders and Schaefer, 2014; Suárez-Álvarez et al., 2016). However, whether pyelonephritic scars contribute to chronic kidney disease (CKD) and the mechanisms involved are unknown (Salo et al., 2011; Toffolo et al., 2012; Nevéus, 2013). Furthermore, it is unclear whether the location, severity or timing of renal fibrosis influences progression to CKD.

An understanding of the link between infection-related fibrosis and subsequent development of CKD has been hindered largely by the lack of robust murine models of ascending severe upper-tract UTI in immunocompetent hosts (Hopkins et al., 1998; Svensson et al., 2005, 2011; Hannan et al., 2010). Although cystitis can be induced by transurethral catheterization in females of many mouse strains, most are resistant to severe pyelonephritis with abscess formation after bladder inoculation (Hopkins et al., 1998; Hannan et al., 2010; Tittel et al., 2011; Hains et al., 2014; Schwartz et al., 2015; Olson et al., 2016). Several previous studies have utilized direct injection of uropathogens into the kidneys (Miller and Phillips, 1981; Santos et al., 1994; Mussalli et al., 1999), but these models bypass ascension of the ureter and clearly do not recapitulate natural arrival of uropathogenic *Escherichia coli* (UPEC) in the collecting system. Other reports have induced female murine pyelonephritis with serial, high-colony-forming-unit (CFU) transurethral inoculations, but did not note gross abscess or severe nephropathy (Tittel et al., 2011; Bowen et al., 2013; Hains et al., 2014; Schwartz et al., 2015). Substantial recent work has been performed in C3H/HeN mice, which are recognized to feature vesicoureteral reflux (Hopkins et al., 1998; Bowen et al., 2013), reflecting a primary risk factor for upper-tract UTI in children (Feld and Mattoo, 2010; Hoberman et al., 2014). In the C3H/HeN mouse strain, a minority of females (the sex historically used almost exclusively in preclinical UTI work because the bladders of male mice cannot reliably be accessed by catheter) develop pyelonephritis – without abscess formation – following bladder inoculation with UPEC, whereas most females resolve infection (Hannan et al., 2010). We previously found, using a novel mini-surgical bladder inoculation technique, that male C3H/HeN mice, unlike females, develop nearly 100% penetrant severe pyelonephritis and renal abscesses following ascending infection, and fail to spontaneously resolve UTI (Olson et al., 2016). Furthermore, infected males exhibit fibrosis and progressive renal disease during later stages of infection (Olson et al., 2016).

In addition, prior studies have examined the generation of inflammation and fibrosis only during untreated, active infection (Svensson et al., 2005, 2011; Bahat Özdoğan et al., 2014; Olson et al., 2016; Li et al., 2017), whereas human patients with pyelonephritis typically would receive antibiotic treatment, such as a cephalosporin or fluoroquinolone, upon recognition of symptoms and appropriate laboratory testing (Warren et al., 1999; Gupta et al., 2011). Thus, we here extended our new preclinical model of UTI in C3H/HeN males to test the efficacy of antibiotic treatment in severe pyelonephritis and in early or established renal abscesses, and to examine long-term sequelae of infection following antimicrobial treatment.

## RESULTS

### Ceftriaxone achieves microbiological cure of pyelonephritis in C3H/HeN males

**Fig. 1.**
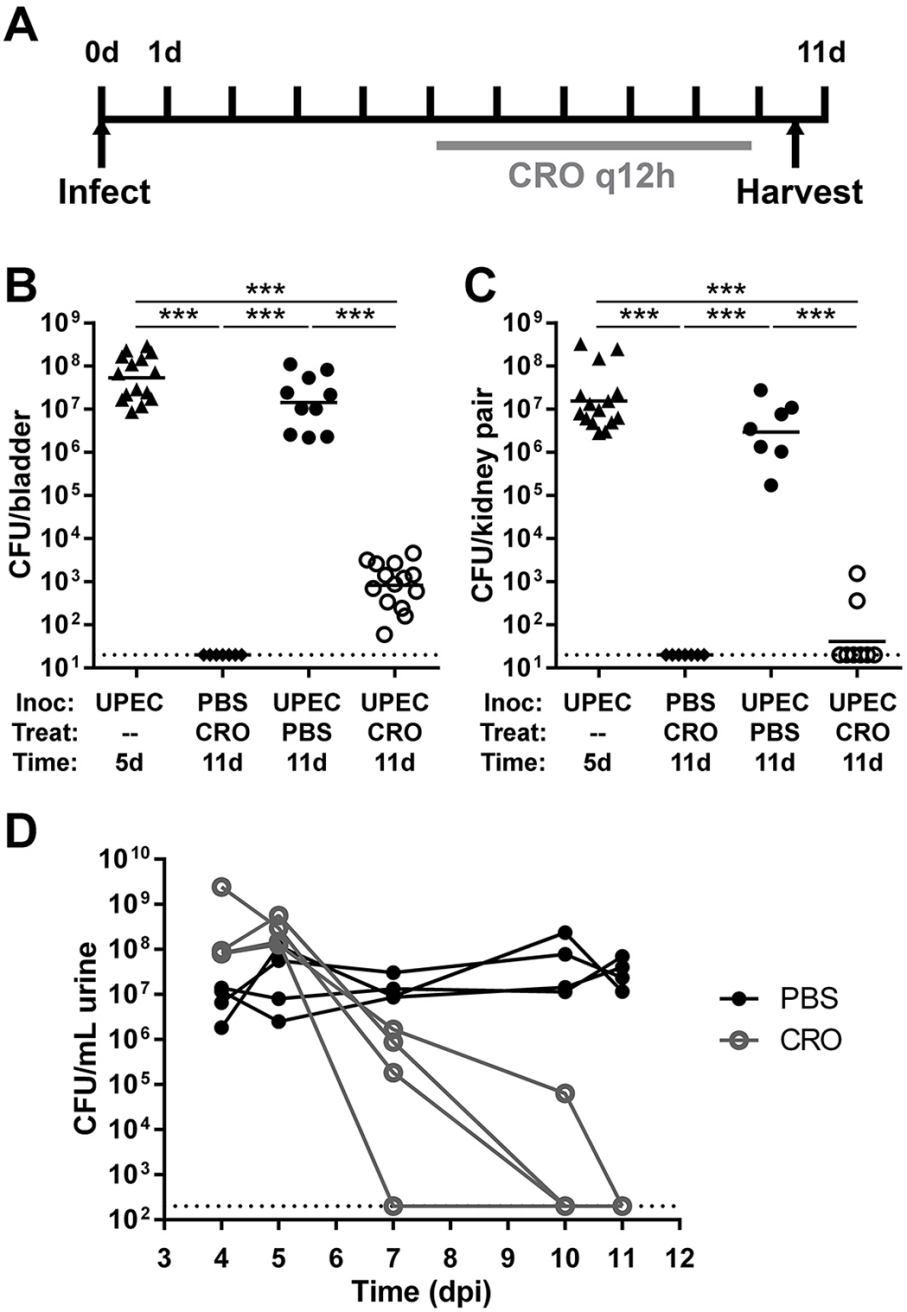
**CRO treatment eliminates renal bacterial burden in C3H/HeN mice with pyelonephritis.** (A) Male C3H/HeN mice were surgically infected with UTI89 or PBS and then treated with CRO or PBS for 5 days starting at 5 dpi. Bladders (B) or kidneys (C) were aseptically homogenized and plated to enumerate CFU 24 h after the last CRO injection. Organ titers in 5-dpi start-of-treatment controls (triangles) were equivalent to mock-treated mice at 11 dpi (black circles), whereas CRO treatment (white circles) significantly reduced bladder bacterial loads and sterilized the infected kidneys. Mock-infected mice (diamonds), as expected, bore no bacteria in bladders or kidneys. Data shown reflect the aggregate of three independent experiments (total *n*=7-15 per condition; ***P<0.001 by Mann–Whitney *U*-test). Dashed lines in all panels represent the limit of detection; bars indicate the geometric mean. (D) Male C3H/HeN mice were surgically infected with UTI89, then either mock-treated (with PBS; black circles) or treated with CRO (gray circles) starting at 5 dpi. Urine bacterial titers on the indicated days are shown. Solid lines connect corresponding urine titers from each individual mouse.

There exist no optimal preclinical models of antibiotic-treated severe pyelonephritis and the immediate or long-term detrimental sequelae of disease. Therefore, we employed mini-surgical inoculation of the bladders of male C3H/HeN mice to model the resolution and sequelae of severe pyelonephritis. By 14 days post-infection (dpi) with UPEC strain UTI89, over 90% of surgically infected C3H/HeN males develop grossly evident, bilateral renal abscess (Olson et al., 2016). In our efforts to model antibiotic treatment, we first attempted multiple ceftriaxone (CRO) dosing schemes starting 14 dpi in male C3H/HeN mice; these strategies failed to effectively treat the advanced abscesses established in kidneys by that time point (Fig. S1). Furthermore, we felt it likely that patients would more commonly present earlier in the course of pyelonephritis. In C3H/HeN males, abscesses rapidly become evident between 5 and 6 dpi, and are fully formed in nearly 100% of males by 7 dpi (Olson et al., 2016; P.D.O., L.K.M. and D.A.H., unpublished data). Therefore, we next elected to initiate CRO or placebo [phosphate-buffered saline (PBS)] administration [given by subcutaneous injection every 12 h (q12 h) for 5 days] beginning at 5 dpi, and harvesting organs upon euthanasia 24 h after the final CRO dose (i.e. 11 dpi; Fig. 1A). Bladder inoculation with UPEC in male C3H/HeN mice resulted in robust bladder (Fig. 1B) and kidney (Fig. 1C) infection in both start-of-treatment controls (5 dpi) and mock-treated animals. CRO treatment significantly reduced bladder (Fig. 1B; *P<*0.0001) and kidney (Fig. 1C; *P<*0.0001) bacterial burdens compared to mock-treated animals. CRO-treated mice continued to harbor 10^2^-10^4^ colony-forming units (CFU) of UPEC in their bladders (Fig. 1B), despite resolving bacteriuria (Fig. 1D); this finding is consistent with prior reports of UPEC reservoirs persisting within bladder tissue following antibiotic treatment (Mysorekar and Hultgren, 2006; Hannan et al., 2010; Blango et al., 2014; Olson et al., 2016). The majority of CRO-treated mice completely resolved kidney infection (Fig. 1C). Trials of other treatment regimens beginning at 5 dpi, including increased CRO duration, dose or frequency, did not further affect organ bacterial burdens (Fig. S2) compared to the 5-day q12 h regimen.

### CRO treatment beginning at 5 dpi sterilizes existing abscesses and halts further abscess formation

**Fig. 2.**
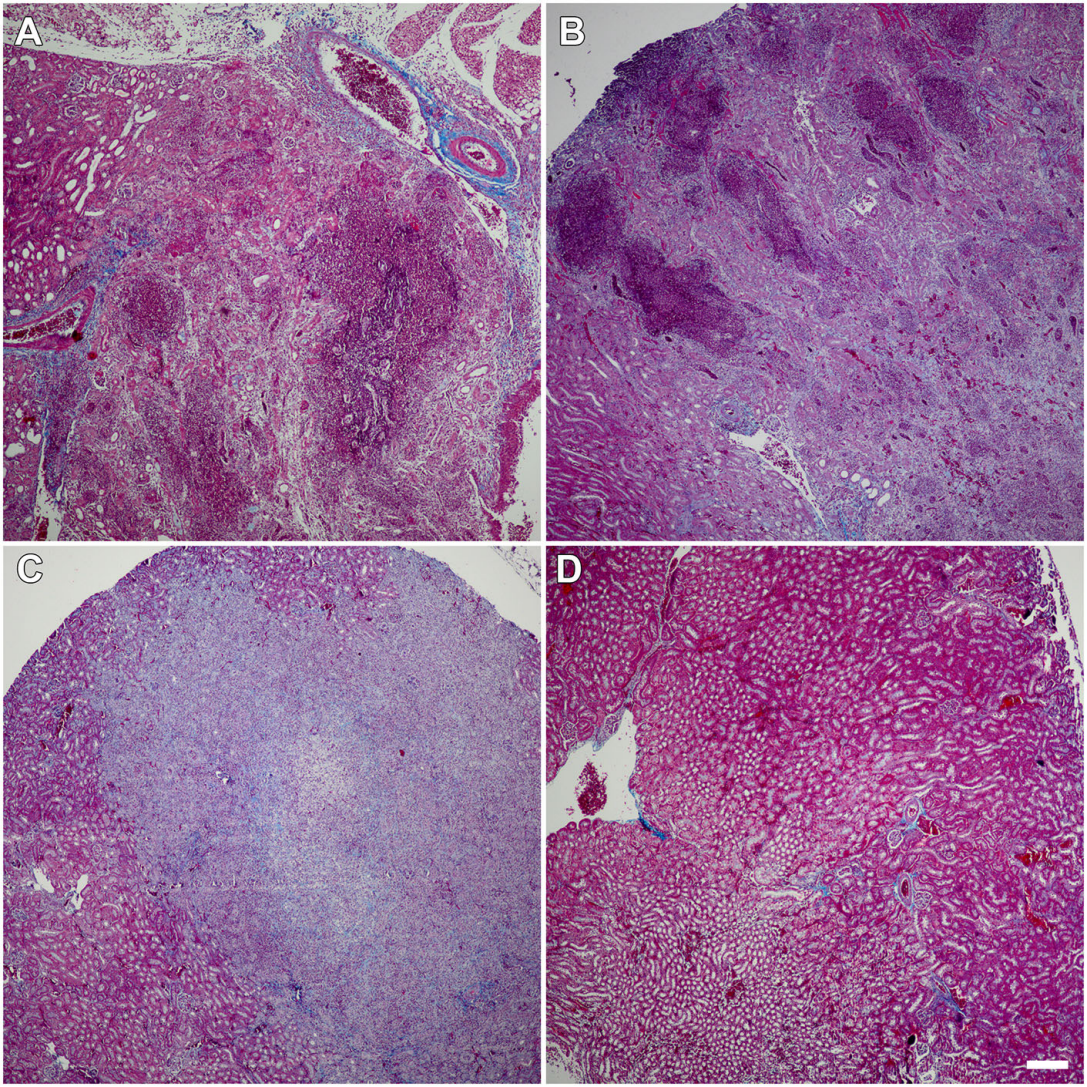
**CRO treatment reduces prevalence of renal abscess.** (A) A minority (27%) of infected C3H/HeN males sacrificed before the start of treatment already exhibited abscess formation at this time point (representative image among *n*=15 mice). (B) 100% of UTI89-infected C3H/HeN males receiving mock treatment (PBS) displayed abscesses 1 day post-treatment (representative image among *n*=10 mice). (C) The prevalence of abscess in UTI89-infected CRO-treated mice (29%) matched that prior to treatment, indicating that CRO treatment prevented further abscess formation but did not reverse tissue damage already present in abscessed kidneys, even though microbiological cure was achieved (representative image among *n*=14 mice). (D) Mock-infected CRO-treated control mice exhibited normal kidney architecture 1 day after treatment conclusion (representative image among *n*=7 mice). Gomori trichrome staining; scale bar: 200 µm.

Among mice sacrificed at the start of treatment (5 dpi), all of which had high kidney bacterial burdens (Fig. 1B), a minority (4 of 15; 27%) demonstrated grossly and microscopically evident abscess formation (Fig. 2A), matching our previous report at the same time point (Olson et al., 2016). By 11 dpi (24 h post-treatment-completion), all (10 of 10) UPEC-infected, mock-treated males displayed gross renal abscess formation (Fig. 2B). Thus, abscess development was progressive during this 6-day interval in the absence of antibiotic treatment. In contrast, the abscess frequency observed at 11 dpi in CRO-treated mice (4 of 14; 29%; *P*=0.0006 versus mock-treated; Fig. 2C) was equivalent to 5-dpi start-of-treatment controls, illustrating that timely antibiotic therapy interrupted abscess progression. As noted above, these CRO-treated mice with evident abscess formation exhibited low kidney bacterial burdens (at or near the lower limit of detection; Fig. 1A) and did not have ongoing bacteriuria (Fig. 1D). These data suggest that CRO treatment beginning at 5 dpi arrested renal abscess development and neutralized the burgeoning UPEC population within the renal parenchyma. As expected, control mice (mock-infected with PBS and treated with CRO) displayed healthy kidney architecture 24 h post-treatment (Fig. 2D).

### Convalescent outcomes in treated pyelonephritis

**Fig. 3.**
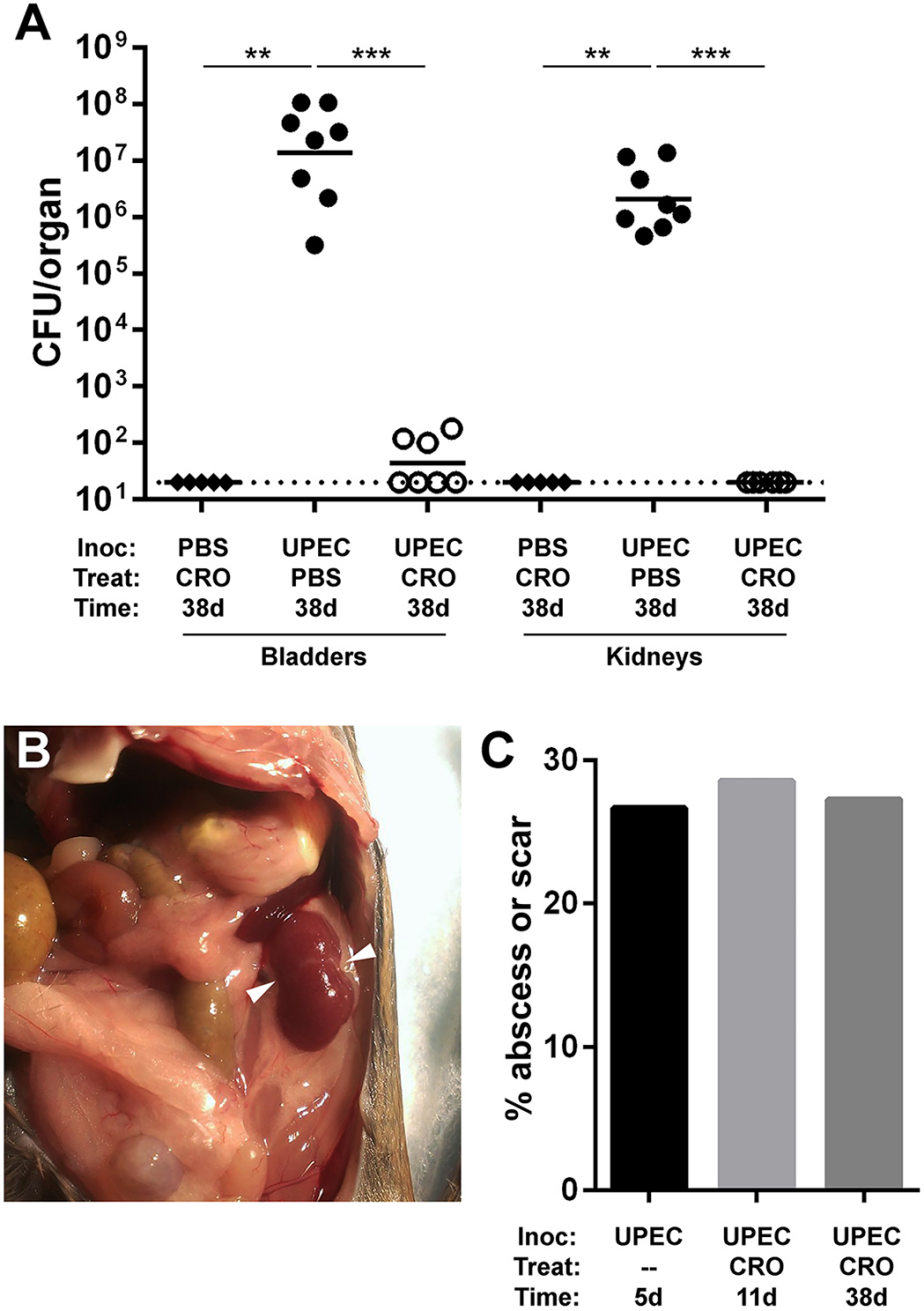
**Renal scars develop following durably successful CRO treatment.** Male C3H/HeN mice were surgically infected (Inoc) with PBS or UTI89 and then treated (Treat) with PBS or CRO. (A) Bladders and kidneys were aseptically harvested, homogenized and CFU enumerated at 38 dpi (28 days post-treatment). CRO treatment significantly reduced bacterial burden and resulted in sterile kidney titers at 38 dpi (aggregate of two experiments, total *n*=5-8 per condition; **P<0.01, ***P<0.001 by Mann–Whitney *U*-test). (B) Following autopsy, gross renal scars were observed in 27% of UPEC-infected CRO-treated mice at 38 dpi. A representative image of a scarred left kidney is shown. Arrowheads indicate gross renal scars. (C) The percentage of animals exhibiting abscesses at 5 dpi (pre-CRO treatment; *n*=15) or 11 dpi (24 h following CRO treatment; *n*=14) is matched by the percentage exhibiting renal scars at 4 weeks post-CRO-treatment (*n*=11) (*P*=not significant by Fisher exact test for each pairwise comparison).

Whereas the majority of CRO-treated mice demonstrated microbiological cure of pyelonephritis 1 day post-treatment, a few maintained very low residual UPEC burdens (Fig. 1B,C). It was unclear whether such UPEC remaining in the bladder or kidney post-CRO-treatment would reemerge to cause recrudescent infection. To specify outcomes in treated pyelonephritis, we treated UPEC-infected mice with CRO or PBS for 5 days, beginning at 5 dpi, and quantified organ bacterial burdens 4 weeks post-treatment. Mock-treated mice all exhibited high bladder bacterial burdens (typical of chronic cystitis) and kidney burdens at this later time point (Fig. 3A), consistent with the near-complete prevalence of these severe UTI outcomes in C3H/HeN males that we reported previously (Olson et al., 2016). All CRO-treated mice resolved renal and bladder infection (Fig. 3A; *P*=0.0007 and *P*=0.0003, respectively, versus mock-treated controls). No CRO-treated mice displayed urine UPEC titers >10^4^ CFU ml^−1^ at biweekly samplings (Fig. S3), but a minority maintained low-level colonization of the bladder (Fig. 3A), again consistent with quiescent reservoir formation as previously reported (Mysorekar and Hultgren, 2006; Hannan et al., 2010; Blango et al., 2014; Olson et al., 2016). Remarkably, gross renal scars were found in several CRO-treated mice at necropsy 4 weeks post-treatment (Fig. 3B, arrowheads). Affected kidneys demonstrated broad-based, U-shaped cortical scarring with retraction of the renal parenchyma, matching the pathological descriptions of human pyelonephritic scars (Smith, 1962; Paueksakon and Fogo, 2014). The fraction of mice displaying grossly visible renal scars 4 weeks following CRO treatment (28%) was equivalent to the fraction of mice demonstrating abscess at either the start of treatment (5 dpi) or 1 day post-treatment (11 dpi) (Fig. 3C). Collectively, these data indicate that the tissue destruction associated with microscopic abscess formation is spatially and pathologically associated with the subsequent development of renal scars.

### Renal scars, despite resolution of infection, harbor progressive inflammation

**Fig. 4.**
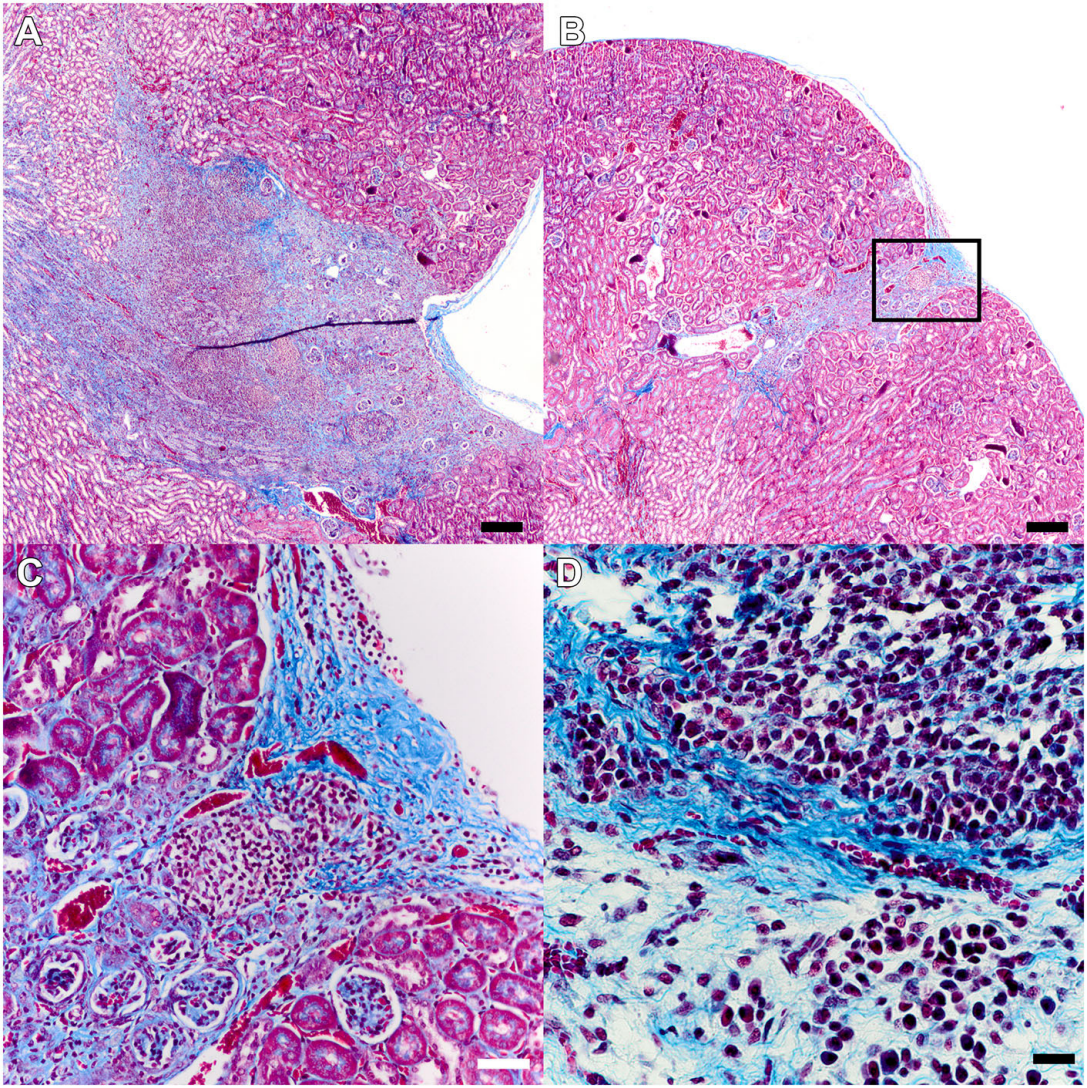
**Post-pyelonephritic scars demonstrate ongoing active inflammation.** Gomori trichrome staining of UPEC-infected mice (*n*=11) with grossly evident scars but negative organ bacterial titers 28 days after completion of CRO treatment demonstrated collagen deposition as well as replacement and retraction of cortical tissue (A,B; scale bars: 200 µm). This was accompanied by dramatic capsular thickening over the scar and a cellular infiltrate (C, inset from B; scale bar: 50 µm) that morphologically consisted primarily of lymphocytes (D; scale bar: 20 µm).

Histopathological analysis of UPEC-infected CRO-treated kidney sections by Gomori trichrome staining revealed extensive cortical scars 4 weeks post-treatment, with collagen deposition in some scars extending from the renal capsule to the medulla (Fig. 4A). The strictures we observed on gross inspection of recovered kidneys (Fig. 3B) were also evident microscopically, and the renal capsule was substantially thickened overlying the scar (Fig. 4B). Fibrosis in these scars followed patterns similar to those observed at earlier stages of abscess development at 5 and 11 dpi in infected CRO-treated mice (see Fig. 2). No scars were observed in mock-infected CRO-treated animals. More striking was the presence of a cellular infiltrate within the scar (Fig. 4C,D), despite all tested animals resolving renal infection (Fig. 3A) and exhibiting sterile urine cultures. Collections of inflammatory cells (primarily lymphocytes) and fibroblasts were embedded within the area of fibrosis (Fig. 4D). These data indicate that the development and maturation of renal scars is an active process that continues following successful microbiological cure of infection with antibiotics.

### Successful CRO treatment restores renal function

**Fig. 5.**
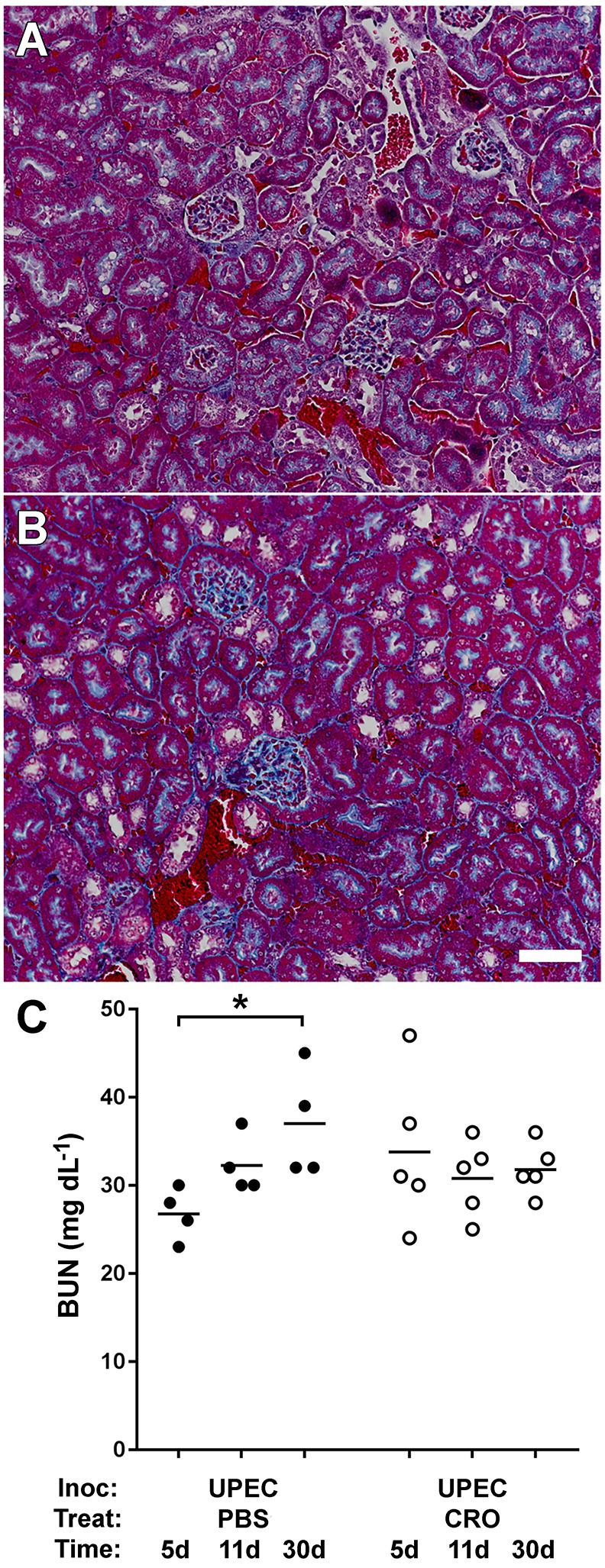
**CRO treatment preserves normal short-term renal function.** (A) Gomori trichrome staining of renal cortex illustrates healthy kidney histology, at 28 days post-treatment, in mock-infected mice treated with CRO (representative image from *n*=10 mice). (B) UPEC-infected mice that resolved pyelonephritis via CRO treatment and lacked gross scars demonstrated minor glomerular sclerosis, but displayed otherwise normal kidney architecture (representative image from *n*=14 mice). Scale bar for A and B: 50 µm. (C) Blood urea nitrogen (BUN) rose over a 30-day interval in UPEC-infected mice (*n*=4-5 per condition) that did not receive antibiotics (*P=0.029 by Mann–Whitney *U*-test, 5 days versus 30 days), but remained at baseline in mice that were treated with CRO (*P*= not significant by Mann–Whitney *U*-test, 5 days versus 30 days).

Human patients that develop acute pyelonephritis typically manifest baseline renal function following resolution of infection; renal scars and other adverse sequelae of resolved infection are identified in a minority of patients, and some are not evident clinically until later in life (Jacobson et al., 1989; Martinell et al., 1996; Wennerström et al., 2000; Shaikh et al., 2010; Levey and Coresh, 2012). In the UPEC-infected CRO-treated mice that resolved infection and lacked evidence of renal scarring 4 weeks post-treatment, histopathological analysis of Gomori trichrome-stained kidney sections showed no increase in interstitial fibrosis compared to mock-infected controls, and normal overall renal architecture similar to mock-infected animals (Fig. 5A,B). However, we frequently observed areas of glomerular sclerosis in these UTI89-infected CRO-treated scar-free animals (Fig. 5B). We also performed serial measurements of blood urea nitrogen (BUN) as a biochemical marker of renal function in UPEC-infected mice. These data demonstrate that BUN remained stable in mice receiving CRO treatment, whereas ongoing infection (i.e. mock treatment) was associated with an increase in BUN (Fig. 5C).

### Long-term outcomes reflect those observed in human patients

**Fig. 6.**
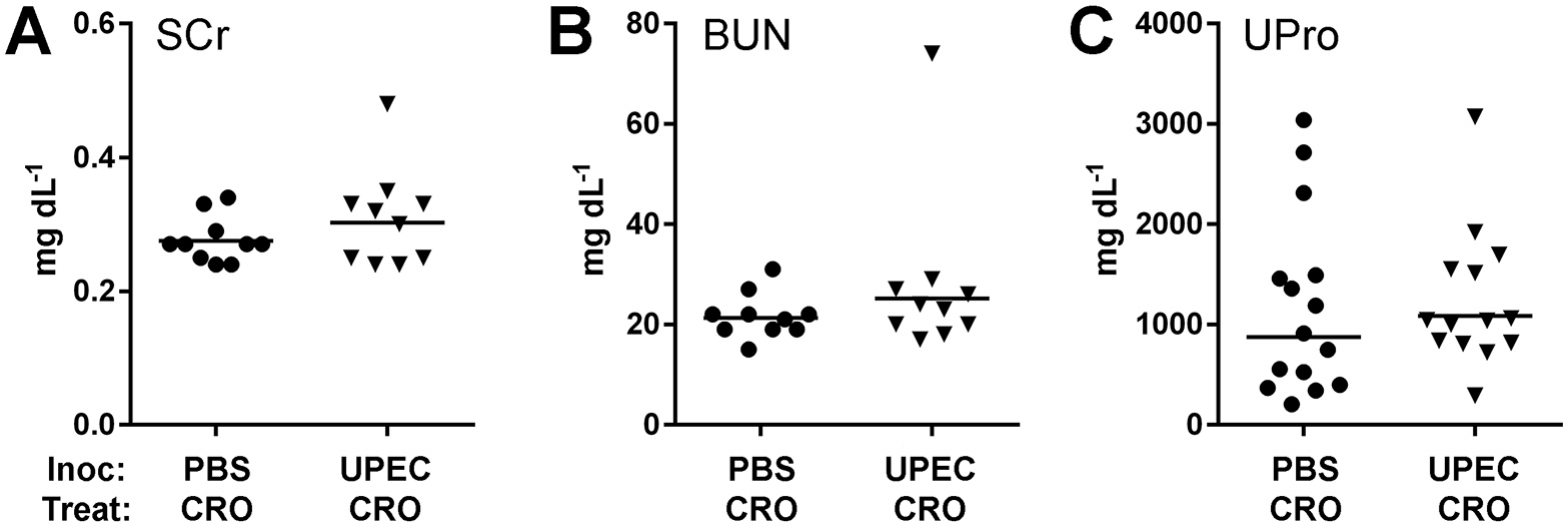
**Successful CRO treatment preserves long-term renal function in most UPEC-infected mice.** Mock-infected mice (PBS; circles) and UPEC-infected mice (triangles) were treated with CRO for 5 days beginning at 5 dpi and aged to 30 weeks (two to three experiments with total *n*=10-15 per condition). Serum was analyzed for creatinine (SCr; A) and blood urea nitrogen (BUN; B), and urine protein was measured (UPro; C). Most UPEC-infected mice demonstrated normal serum creatinine, BUN and urine protein at 30 weeks of age (*P*= not significant by Mann–Whitney *U*-test for each analyte), although one UPEC-infected mouse that displayed notable bilateral scars also had marked elevation in these biochemical markers.

Children who develop renal scars following UTI may be followed into adulthood, when signs of CKD may manifest in a minority of such patients (Jacobson et al., 1989; Martinell et al., 1996; Wennerström et al., 2000; Levey and Coresh, 2012). Therefore, we surgically inoculated male C3H/HeN mice with either PBS (mock) or UTI89, treated with CRO for 5 days beginning at 5 dpi and then observed these mice until 30 weeks of age (i.e. 5 months post-treatment). Successful antibiotic treatment preserved normal renal function in the majority of UPEC-infected mice at this longer interval; mean serum creatinine (Fig. 6A), BUN (Fig. 6B) and urine protein (Fig. 6C) were not significantly higher than in mock-infected mice. However, in one UPEC-infected mouse (Fig. 6) that developed particularly notable bilateral scars, these three biochemical markers were markedly higher, indicating the development of CKD.

**Fig. 7.**
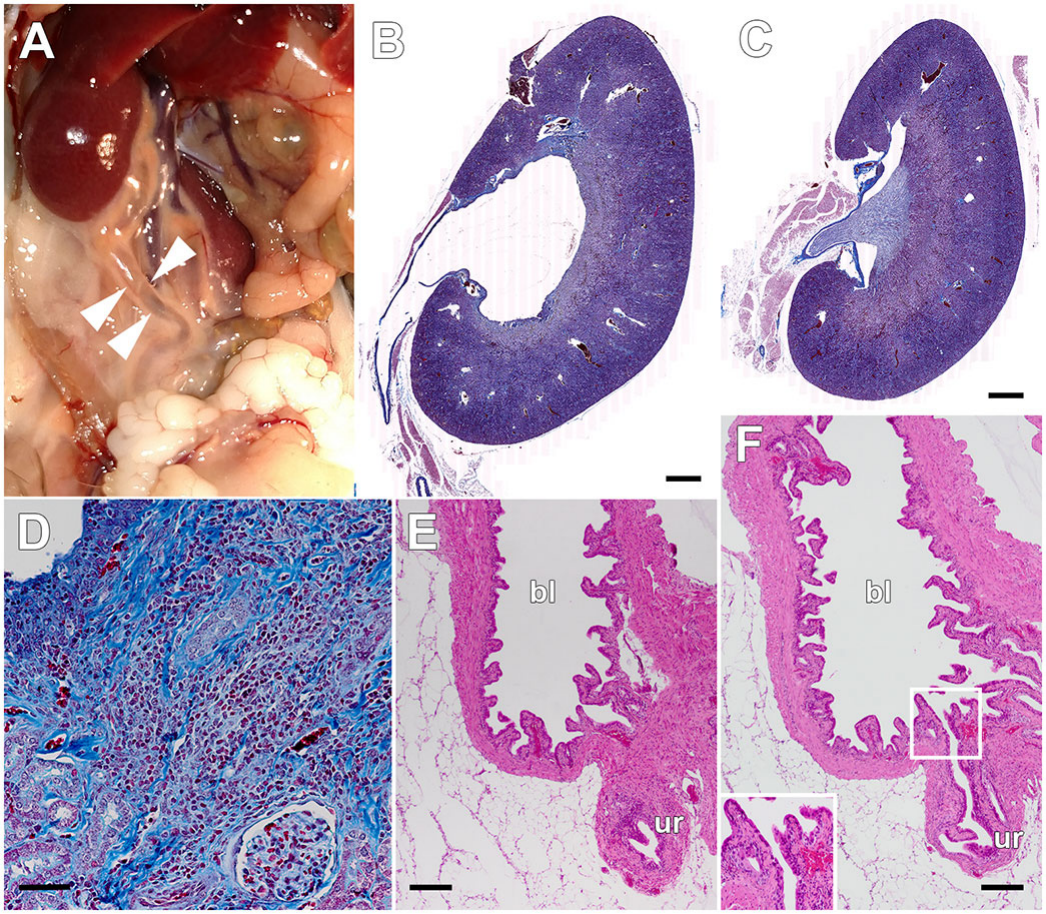
**Hydronephrosis is observed 5 months post-successful-treatment.** Male C3H/HeN mice were treated with CRO for 5 days beginning at 5 dpi and aged to 30 weeks as in Fig. 6. The majority of UPEC-infected CRO-treated males exhibited grossly visible hydronephrosis with dilated ureters (A, arrowheads). Histopathology of Gomori trichrome-stained sections confirmed dilated collecting structures and proximal ureter in UPEC-infected CRO-treated mice (B; scale bar: 500 µm), whereas mock-infected CRO-treated mice displayed normal kidney architecture without discernible hydronephrosis (C; scale bar: 500 µm). As was also noted at 4 weeks post-treatment, UPEC-infected CRO-treated animals at 30 weeks of age displayed fibrotic scars and continued active inflammation, despite resolving infection (D; scale bar: 50 µm). Complete sectioning of the bladders from these animals (E and F; scale bars: 200 µm) did not reveal evidence of obstruction at the ureterovesical junction; E shows the ureter (ur) as it approaches the wall of the bladder (bl), and F is a serial section showing patency of the ureterovesical junction (magnified in inset). Images are representative of two experiments, total *n*=10 mice per condition.

Surprisingly, we found grossly visible bilateral hydronephrosis in 80% of the UPEC-infected CRO-treated mice allowed to age to 30 weeks. These animals had bilaterally dilated ureters (Fig. 7A), and expansion of the renal pelvis was visible on bisection of the kidneys. Histopathology confirmed these findings, with infected animals displaying enlarged, dilated renal pelvis and calyces, flattening of the pelvic epithelia, atrophy and thinning of the renal cortex, and expanded ureters (Fig. 7B). These abnormal features were uniformly absent in mock-infected CRO-treated animals at 30 weeks of age (Fig. 7C). There was no evidence of hydronephrosis at 11 or 38 dpi in any mock- or UPEC-infected mock- or CRO-treated mice (Figs 2, 3, 4). Fibrotic scars in the parenchyma near the renal pelvis, with persistent inflammatory infiltrates, were evident microscopically at 30 weeks of age in UPEC-infected CRO-treated mice (Fig. 7D, as seen at 38 dpi), but not in mock-infected controls. Histological examination of the bladders of UPEC-infected CRO-treated mice at this long time point revealed epithelial changes reflecting bladder remodeling and chronic inflammatory infiltrates, but no evidence of obstructive lesions at the ureterovesical junction (Fig. 7E,F).

## DISCUSSION

Here, we developed a new model to enable preclinical studies of the resolution and sequelae of antibiotic-treated upper-tract UTI. To do so, we leveraged the mini-surgical inoculation technique that allows infection of C3H/HeN males, which develop nearly 100% penetrant severe pyelonephritis and renal abscess following bladder inoculation with UPEC. Although antibiotic treatment alone was not successful when initiated at later time points (presumably because of very advanced infection, and consistent with clinical experience in patients with established abscesses), CRO treatment initiated at 5 dpi achieved resolution of infection, aborting the abscess development that begins at that time. An appropriate minority of these infected and successfully treated animals developed renal scars by 1 month post-treatment; these scars showed fibrosis and ongoing inflammatory cellular infiltrates. At longer follow-up, an even smaller proportion demonstrated biochemical evidence of CKD.

Roughly one third of infected male C3H/HeN mice exhibited abscess at 5 dpi (at the start of treatment) or demonstrated sterile abscess post-treatment, and a similar fraction of mice demonstrated renal scarring 1 month after successful antibiotic treatment. Current limitations of live-animal imaging preclude a definitive link between the anatomic locations of initial abscess and ultimate renal scar. However, we can reasonably posit that abscess development, with associated renal parenchymal necrosis and replacement with inflammatory infiltrates, gives way to scar formation in this spatially identical region of a given kidney. Following antibiotic treatment, sterile abscesses featured inflammation and tissue destruction similar to descriptions of active abscess at 5 dpi (Fig. 2), and inflammation persisted in renal scars at later time points (Fig. 5), consistent with reports from human pathology (Bernstein and Arant, 1992). This temporal and spatial association argues that the immune responses to UPEC introduction and the inflammatory processes surrounding micro- and macroabscess formation lay the mechanistic foundation for scar development. This hypothesis is also supported by recent findings in the C3H/HeOuJ mouse strain (Li et al., 2017). It follows that, if these mechanisms can be understood at a molecular level, future targeted therapeutic modalities may attenuate or alter the nature of renal inflammation and/or impact the inflammatory modulators released from pyelonephritic scars (Haraoka et al., 1994; Pohl et al., 1999; Nevéus, 2013; Bahat Özdoğan et al., 2014), ultimately reducing risk for CKD.

Our preclinical model of renal scarring following successful antibiotic treatment of ascending upper-tract UTI fills a substantial gap in the field and reproduces the outcomes observed in patients, particularly children, with pyelonephritis. Only a small percentage of humans presenting with upper-tract UTI develop renal scars following resolution of acute infection, and it is unclear why some individuals develop these scars whereas others do not (Hewitson, 2009; Shaikh et al., 2010; Strohmeier et al., 2014). Estimates of the risk of renal scarring after pyelonephritis in children vary, but range between 8 and 40%, with a meta-analysis concluding that ∼15% of such children have demonstrable evidence of scarring at follow up (Jakobsson et al., 1994; Shaikh et al., 2010). The model described in the present work approximates this proportion, with 27-29% of mice developing renal scars. Clinical studies have shown that early and aggressive antibiotic treatment minimizes the risk of renal scar formation (Miller and Phillips, 1981; Winter et al., 1983; Shaikh et al., 2016); our studies reinforce this point, suggesting that minor delays in the start of antimicrobial treatment could substantially influence whether permanent renal damage occurs or whether pyelonephritis instead resolves without complication.

This work was limited to the study of an adult male murine model, employed to overcome the shortcomings of previous female models of pyelonephritis. The sex ratio in UTI among infants favors females, but approximates 2:1 over the first 2 years of life. A number of studies indicate that male cases outnumber female neonatal UTI within the first 6 months after birth (Winberg et al., 1974; Ginsburg and McCracken, 1982; Wettergren et al., 1985; Kanellopoulos et al., 2006; Wong et al., 2010; Ismaili et al., 2011; Park et al., 2011; Bonadio and Maida, 2014). Furthermore, several reports suggest that male sex may be a prognostic factor for the development of renal scarring in infants that develop febrile UTI (Marra et al., 2004; Soylu et al., 2008; Mattoo et al., 2015). This matches a growing body of evidence that male sex may be an indicator for worse morbidity, mortality or sequelae from pyelonephritis (Nicolle et al., 1996; Efstathiou et al., 2003; Foxman et al., 2003; Ki et al., 2004; Olson et al., 2016). Additionally, the clinical data supporting an age-related influence on risk for scarring after childhood pyelonephritis are variable, but on balance may indicate a slight predisposition for developing renal scars in older children presenting with pyelonephritis (Benador et al., 1997; Ataei et al., 2005; Shaikh et al., 2014). Future work with our CRO treatment model in female models of ascending UTI and in male mice of varying age could further define the influences of sex and age, respectively, on post-pyelonephritic fibrosis and sequelae of infection.

Our experiments also reveal translationally appropriate rates of long-term sequelae, both scarring and CKD. To clearly delineate the association and mechanistic pathways leading to CKD specifically in this model, future studies will require substantially larger sample sizes. Alternatively, the incidence of the CKD outcome might be augmented by infecting with a greater inoculum or by initiating treatment somewhat later in infection (e.g. 7 dpi), when a larger proportion of C3H/HeN males will have established abscesses (Olson et al., 2016). Most non-infectious murine models of CKD require some combination of unilateral or subtotal nephrectomy, ureteral obstruction, and injury or insult to the remaining kidney (Davies et al., 2003, 2005; Manson et al., 2011; Agapova et al., 2016); one could imagine attempting to increase the incidence of post-pyelonephritic CKD by introducing a similar unilateral or partial nephrectomy procedure prior to UPEC infection and antibiotic treatment. Measurement of blood pressure will help to correlate renal scars in mice with hypertension, which is more common as a sequela of human pyelonephritis than is CKD. Continued work along these lines will provide further evidence to define the relationship between pyelonephritic scar formation and risk for CKD.

Although it is accepted that UTI risk is enhanced in individuals with hydronephrosis associated with vesicoureteral reflux (VUR) or urodynamic obstruction (Feld and Mattoo, 2010; Hoberman et al., 2014), there is, to our knowledge, no conclusive paradigm in which pyelonephritis and/or severe cystitis causes or reveals the presence of hydronephrosis. Several early studies did speculate that infection might cause an increase in VUR, although the evidence for cystitis either promoting VUR or having no effect on VUR is relatively weak (Hanley, 1962; Howerton and Lich, 1963; Tanagho et al., 1965; Gross and Lebowitz, 1981; Garin et al., 1998). The hydronephrosis we observed in long-term follow-up of infected and successfully treated C3H/HeN males – mice known to have preexisting VUR – suggests that incident UTI may worsen such reflux, either temporarily or in a more protracted way. Future studies that optimize models of severe pyelonephritis in non-refluxing backgrounds such as C57BL/6 (Tittel et al., 2011; Hains et al., 2014) may ascertain the contribution of VUR to the phenotypes we observed in C3H/HeN males. Other potential causes of bilateral hydronephrosis in these recovered males, such as bilateral ureteral or ureterovesical obstruction, were not observed but are not completely excluded by the present data. Papillary blunting and hydronephrosis were not seen in UPEC-infected, mock-treated animals, or at earlier time points immediately following antibiotic treatment, suggesting that these findings may arise in association with long-term remodeling and fibrosis responses in the bladder, ureter and/or kidney. In any case, fibrosis is evident much earlier in the course of recovery than is hydronephrosis; it is therefore unlikely that hydronephrotic injury is the initiator of fibrosis in this model, although it may contribute to progression of fibrosis. Other recent studies have begun to illuminate not only macroscopic, but microscopic and molecular, imprints left by severe UTI on the urinary tract and its cellular constituents (O'Brien et al., 2015, 2016; Schwartz et al., 2015). These studies may support the concept of a vicious cycle in patients with urodynamic abnormalities, who are already predisposed to UTI but whose urodynamics may also regress with repeated UTI.

In summary, we report a novel system for modeling the complications arising from severe UTI. Susceptible hosts develop renal abscess upon ascending UPEC infection and, despite successful antibiotic therapy, a translationally relevant proportion of mice ultimately develop renal scars after treatment. This model promises to address the relationships between the development of pyelonephritis, timing and effectiveness of antibiotic therapy, and sequelae (including renal scarring and CKD), as well as to illuminate the soluble and cellular inflammatory components responsible for ongoing renal damage following microbiological resolution.

## MATERIALS AND METHODS

### Bacteria

UPEC strain UTI89 was isolated from a patient with cystitis (Chen et al., 2009). For surgical infections, bacteria were grown statically in Luria-Bertani (LB) broth for 16 h at 37°C. The cultures were centrifuged for 10 min at 7500 ***g*** at 4°C before resuspension in sterile PBS to a final density of 4×10^8^ CFU ml^−1^.

### Introduction of murine UTI

All animal procedures received prior review and approval from the Institutional Animal Care and Use Committee at Washington University, St. Louis, MO, USA. A widely used female murine model of cystitis with transurethral inoculation via catheter has been described in methodological detail (Mulvey et al., 1998; Hung et al., 2009; Hannan and Hunstad, 2016), but this approach is technically precluded in male animals. A recently developed surgical approach (Olson et al., 2016) was used to initiate infection in male mice. Eight-week-old male C3H/HeN mice (Envigo, Indianapolis, IN) were maintained under inhalation anesthesia with 3% isoflurane via vaporizer and nose cone. Briefly, anesthetized mice were positioned supine, shaved and the ventral abdomen was sterilized with 2% chlorhexidine solution. A vertical, midline incision (3 mm in length) was made directly overlying the bladder, first through the abdominal skin and then through the peritoneum. The bladder was exposed, aseptically emptied and punctured with a 30-gauge 0.5-inch needle adapted to a 1-ml tuberculin syringe containing the bacterial inoculum. Fifty microliters containing 1×10^7^-2×10^7^ CFU was introduced to the bladder lumen over 10 s, the bladder was allowed to expand for a further 10 s, and the needle was then withdrawn. The peritoneum and the skin were closed with simple, interrupted sutures, and the animal was awakened in fresh air.

### CRO treatment

We adapted CRO treatment regimens reported previously in female murine models to clear UTI and to provide circulating drug levels similar to those seen in patients treated with CRO (Lepeule et al., 2012; Tratselas et al., 2014). At 120 h after surgical infection, male C3H/HeN mice received 125 mg kg^−1^ CRO dissolved in sterile water by subcutaneous injection; mock-treated animals received an equivalent volume of PBS. Mice received similar subcutaneous injections of CRO or PBS every 12 h for 5 days (i.e. ten doses in total). Bladders and kidneys were aseptically harvested 24 h after the last treatment to allow for clearance of residual CRO from the tissues (see Fig. 1A).

### Determination of urine and tissue bacterial loads

Where indicated, we noninvasively obtained post-infection clean-catch urine samples using gentle suprapubic pressure for serial dilution and plating to enumerate CFU ml^−1^ urine. At the indicated time points, mice were euthanized via CO_2_ asphyxiation, and bladders and kidney pairs were aseptically removed and homogenized in 1 ml or 0.8 ml sterile PBS, respectively. We plated serial dilutions of tissue homogenates on LB agar to enumerate bacterial loads. Where indicated, infection in C3H/HeN mice was classified as ‘chronic’ if all urine and endpoint bladder titers contained >10^4^ CFU ml^−1^, whereas ‘resolved’ mice demonstrated endpoint bladder burdens and at least one urine time point with <10^4^ CFU ml^−1^ (Hannan et al., 2010).

### Blood and urine chemistries

Serum or urine was analyzed on the day of collection for BUN, serum creatinine or urine protein by standard autoanalyzer laboratory methods performed by the Department of Comparative Medicine veterinary facility at Washington University.

### Tissue histopathology

Infected bladders and kidneys were bisected and fixed in 10% neutral buffered formalin for 24 h. Fixed tissues were embedded in paraffin, sectioned, and stained with hematoxylin and eosin or Gomori trichrome stain before light microscopy. Whole-kidney images (Fig. 7B,C) were acquired with the AxioScan Z1 Automated Slide Scanning System and digitally tiled by the acquisition software.

### Statistical analysis

Statistics and graphing were performed using Prism 7 (GraphPad Software, La Jolla, CA). Organ bacterial loads and other numerical data were compared by the nonparametric Mann–Whitney *U*-test. 2×2 comparisons were analyzed using the Fisher exact test. *P*-values <0.05 were considered significant.

## Supplementary Material

Supplementary information
