# Correction: Renal scar formation and kidney function following antibiotic-treated murine pyelonephritis (doi: 10.1242/dmm.030130)

**DOI:** 10.1242/dmm.036798

**Published:** 2018-09-13

**Authors:** Patrick D. Olson, Lisa K. McLellan, Alice Liu, Kelleigh E. Briden, Kristin M. Tiemann, Allyssa L. Daugherty, Keith A. Hruska, David A. Hunstad

There was an error published in *Dis. Model. Mech.* **10**, 1371-1379 (doi: 10.1242/dmm.030130).

The middle initial of co-author Kelleigh Briden was incorrectly stated as L. rather than E.

The correct listing appears above and the original article has been corrected accordingly.

