# Supplementary material for: Renal scar formation and kidney function following antibiotic-treated murine pyelonephritis
Source: Dis Model Mech. 2017 Nov 1;10(11):1371–9. doi: 10.1242/dmm.030130 (PMC5719254; doi:10.1242/dmm.030130)
Supplement: Supplementary information [file dmm-10-030130-s1.pdf]

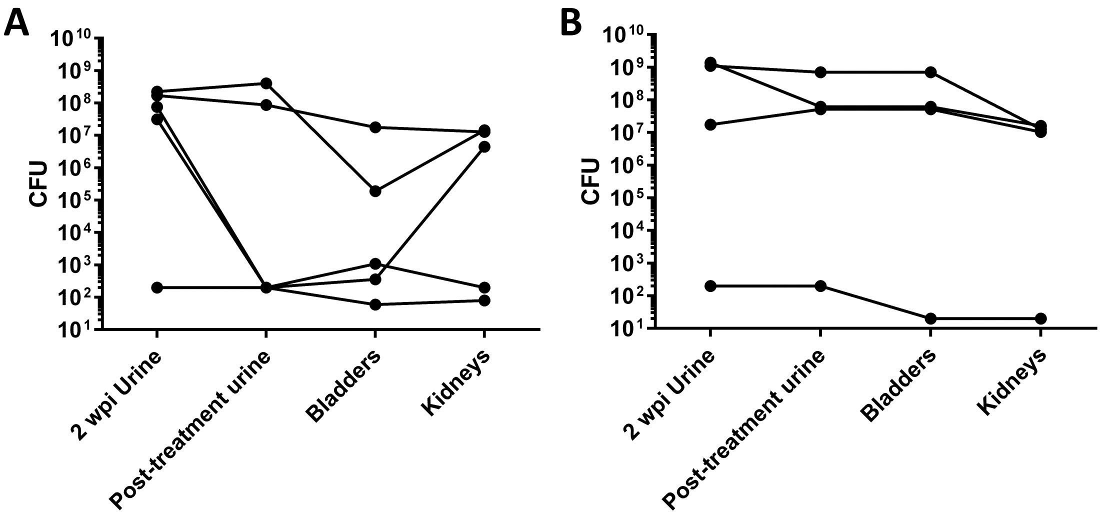

Supplementary Figure S1. Antibiotic treatment begun 2 weeks post infection (wpi) fails to sterilize the urinary tract. Male C3H/HeN mice were infected with UTI89 as described in Materials and Methods. Beginning 2 wpi, mice received ceftriaxone 125 mg/kg SQ every 12 h for 5 d (**A**) or an equivalent volume of PBS (**B**). Shown for each condition are urine bacterial loads (colony-forming units [CFU]/mL) prior to the start of treatment (2 wpi) and at the conclusion of treatment, and the bacterial loads (CFU/organ) in bladders and kidneys upon sacrifice 20 dpi. A solid line connects the data points for each individual mouse.

Disease Models & Mechanisms • Supplementary information

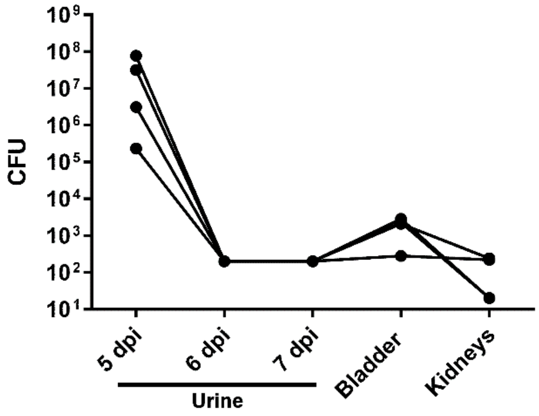

Supplementary Figure S2. Ceftriaxone dosing every 6 h yields microbiologic cure similar to the twice-daily regimen. C3H/HeN male mice were infected with UTI89 as described in Materials and Methods, and ceftriaxone 70 mg/kg SQ every 6 h was commenced 5 dpi. Shown are urine titers (colony-forming units [CFU]/mL) pre-treatment (5 dpi) and at 6 and 7 dpi, as well as bladder and kidney bacterial loads (CFU/organ) at sacrifice 11 dpi. A solid line connects the data points for each individual mouse.

Disease Models & Mechanisms • Supplementary information

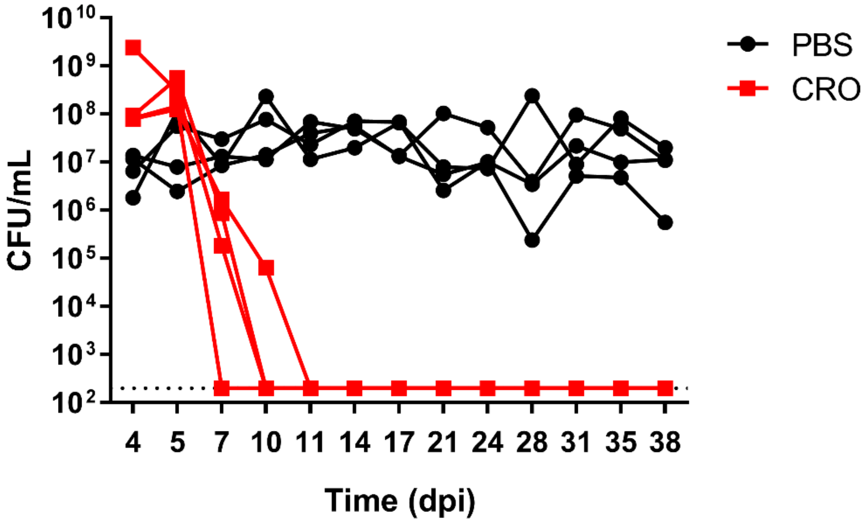

Supplementary Figure S3. Ceftriaxone treatment rapidly sterilizes urine cultures. Shown are bacterial loads (colony-forming units [CFU]/mL) in urine cultures at the indicated time points in UPEC-infected C3H male mice receiving a 5-day course of ceftriaxone (CRO; red) or control (PBS; black) beginning 5 dpi. A solid line connects the data points for each individual mouse.
